# Cross-cultural adaptation and validation of the Chinese version of the Maternal Adaptation Scale: a methodological study

**DOI:** 10.4069/whn.2026.06.03

**Published:** 2026-06-30

**Authors:** Feiyan Yi, Sukhee Ahn

**Affiliations:** College of Nursing, Chungnam National University, Daejeon, Korea

**Keywords:** Adaptation, psychological, Depression, postpartum, Parenting, Social support, Parenting, Validation study

## Abstract

**Purpose:**

This study aimed to translate the Korean version of the Maternal Adaptation Scale into Chinese, develop the Chinese version of the Maternal Adaptation Scale (C-MAS), and evaluate its reliability and validity among Chinese women.

**Methods:**

This cross-sectional methodological study evaluated the reliability and validity of the C-MAS. A total of 500 primiparous and multiparous Chinese women who visited hospitals in Weifang and Hebi, China, for infant vaccination or postpartum care were included. Psychometric evaluation of the C-MAS included confirmatory factor analysis (CFA), correlation analysis, known-groups comparison, and reliability analysis.

**Results:**

CFA provided empirical support for the six-factor model of the C-MAS, and all 26 retained items had factor loadings greater than 0.40. Model fit indices supported the construct validity of the C-MAS, and reliability analyses indicated strong internal consistency across items (Cronbach’s α=.96). Criterion-related validity was supported by significant correlations between maternal adaptation and parenting efficacy (r=.39, *p*<.001), infant-care social support (r=.14, *p*<.001), and postpartum depression (r=−.39, *p*<.001). Known-groups validity was also supported, as women without depressive symptoms had significantly higher maternal adaptation scores than women with depressive symptoms. Measurement invariance testing demonstrated consistent psychometric properties across primiparous and multiparous women, indicating that the scale measured the same construct in both groups.

**Conclusion:**

The C-MAS is a psychometrically sound instrument with good reliability and validity for assessing maternal adaptation among Chinese postpartum women. Its applicability to both primiparous and multiparous women supports its use in postpartum research and clinical practice in China.

## Introduction

The theory of becoming a mother, also known as maternal role attainment, conceptualizes motherhood as a dynamic, developmental, and interactive process that encompasses self-perception, understanding of motherhood, emotional experiences with the infant, social interaction and learning, and ongoing socialization [1]. During the postpartum period, mothers must recover physically, acquire infant care and parenting skills, and adjust to new family and social priorities [2]. Although these experiences reflect the core principles of Mercer’s theory [1], mothers often encounter challenges related to their maternal role and identity, including parenting stress, changing family responsibilities and relationships, and the reorganization of social relationships [3-7]. Maternal adaptation begins in the early postpartum period, and 4 months postpartum represents an important stage in the development of maternal identity and functioning [8]. Factors that influence maternal adaptation include positive factors, such as parenting competence and family social support [9-11], and negative factors, such as postpartum depression [10]. As the responsibilities of motherhood expand and intensify during this period, nurses can support maternal adjustment by addressing key variables associated with maternal adaptation.

The Korean version of the Maternal Adaptation Scale (K-MAS), developed for first-time mothers [12] based on Roy Adaptation Model, assesses maternal adaptation across six domains: role performance ability, role cognition ability, maternal acceptance, physical and physiological recovery, adaptation to family relationships, and adaptation to social relationships. These domains conceptually overlap with constructs measured by Chinese maternal role instruments, such as maternal role well-being, caregiving ability, and maternal beliefs [13,14], while also extending assessment to physical recovery and social adaptation. This broader scope indicates the need for instruments that assess maternal adaptation beyond role performance and can inform nursing interventions. These multidimensional domains also support the need for cross-cultural validation of a culturally sensitive, reliable, and comprehensive assessment tool.

Although maternal adaptation has been studied primarily among primiparous women, maternal role adaptation is theoretically applicable to all mothers because it encompasses ongoing adjustments in maternal roles, identities, and capabilities throughout parenting [1]. However, psychometric evaluation of maternal adaptation instruments among multiparous women remains limited, particularly in the context of China’s changing fertility policies [7,15].

After implementation of the two-child policy in 2016, increasing numbers of women in their 30s and early 40s in China pursued subsequent pregnancies [16]. Multiparous women may experience greater challenges in maternal adaptation than primiparous women because of intensified childcare demands [16,17], heightened psychological stress, and reduced social support [18,19]. Accordingly, assessment of maternal role adaptation and identity formation among all mothers is essential for designing effective nursing interventions.

China and Korea are geographically proximate and share several sociocultural traditions rooted in East Asian values, including strong family involvement in infant care and culturally structured postpartum care practices, such as *zuo yuezi* in China and *sanhujori* in Korea [20-22]. These shared contexts may shape similar expectations regarding maternal roles and caregiving responsibilities [23]. However, differences in healthcare systems, postpartum services, and family structures may also influence maternal adaptation experiences [24]. Therefore, cross-cultural translation and validation are necessary before the Korean measurement tool can be applied to Chinese mothers. This study aimed to translate the K-MAS into Chinese and culturally adapt and validate the resulting instrument for measuring maternal adaptation among Chinese mothers.

## Methods

Ethics statement: This study was approved by the Institutional Review Board of Chungnam National University (No. 202408-SB-105-01). Informed consent was obtained from all participants.

### Study design

This methodological, cross-sectional study evaluated the construct validity and internal consistency of the Chinese version of the Maternal Adaptation Scale (C-MAS) among mothers at 4 to 6 months postpartum. Reporting followed the STROBE (Strengthening the Reporting of Observational Studies in Epidemiology) guidelines.

### Participants

A total of 500 mothers at 4 to 6 months postpartum were recruited during outpatient clinic visits at two tertiary general hospitals in China: Weifang Medical University/Weifang City Maternity and Child Health Center in Shandong Province and the Department of Obstetrics and Gynecology/Pediatrics of Hebi City Maternity and Child Health Center in Henan Province.

Participants were eligible if they were aged 20 years or older; had delivered a full-term singleton infant, defined as a gestational age of 37 to 42 weeks and a birth weight greater than 2.5 kg, without congenital malformations or serious illness; were raising their infant with a spouse; and were able to understand and complete the study questionnaire. Mothers were excluded if they had a serious physical or mental illness during the perinatal period, had delivered a preterm infant, or had an infant who was admitted to a hospital for specialized medical care. According to the recommendations of Terwee et al. [25], the sample size was calculated using a ratio of 10 participants per item for factor analysis. To examine potential differences in factor structure between primiparous and multiparous mothers, 500 participants were recruited, with 250 planned for each group.

### Tool translation procedure

After permission was obtained from the original authors, the first author translated the Korean version of the scale into Chinese [12]. The translation process followed a rigorous multistep procedure to ensure linguistic and cultural fidelity. First, a bilingual Korean-Chinese doctoral nursing student translated the instrument from Korean into Chinese. This Chinese translation was then reviewed by a bilingual Chinese nursing professor fluent in both languages, who provided feedback to improve linguistic clarity and preserve conceptual accuracy. A separate bilingual doctoral nursing student back-translated the instrument into Korean. Another bilingual professor then compared the back-translation with the original instrument to identify discrepancies arising from linguistic or cultural nuances. Appropriate adjustments were recommended to improve semantic alignment. Finally, one author and the translator jointly evaluated the final Chinese version to confirm its equivalence with the original Korean instrument.

Expert content validity was evaluated by two bilingual nursing professors with expertise in maternal health. Each item was rated on a 4-point scale ranging from 1 (not relevant) to 4 (highly relevant), and ratings of 3 or 4 were considered relevant. Expert 1 rated four items as 3 and 24 items as 4, whereas Expert 2 rated six items as 3 and 22 items as 4. All items were rated as relevant by both experts, resulting in an item-level content validity index of 1.00 for all 28 items. The scale-level content validity index based on the average method was also 1.00, indicating excellent content validity.

Finally, five Chinese mothers participated in pilot testing of the translated instrument and provided feedback on clarity, interpretation, and cultural appropriateness. This feedback informed the final revisions, and the resulting instrument was used in the validation study.

### Measurement

#### Maternal adaptation

This study used the C-MAS translated from the K-MAS [12]. The translated C-MAS includes 28 items across six domains: role performance ability, role cognition ability, maternal acceptance, physical and physiological recovery, adaptation to family relationships, and adaptation to social relationships. Each item is rated on a 4-point Likert scale, with total scores ranging from 28 to 112. The overall reliability of the original instrument was 0.87, and reliability for the six subscales ranged from 0.73 to 0.84 [12].

#### Parenting competence

The Chinese version of the Parenting Sense of Competence Scale (C-PSOC) [26,27] was used to assess the criterion validity of the C-MAS. This scale consists of 17 items across two domains: parenting efficacy and parenting satisfaction. Each item is rated on a 6-point scale, with higher scores indicating greater parenting competence. The scale demonstrated a Cronbach’s α of .82 for reliability and a content validity index of .98 in a previous validation study [27]. In the present study, the reliability of the C-PSOC was confirmed with a Cronbach’s α of .94.

#### Postpartum depression

The Chinese version of the Edinburgh Postpartum Depression Scale (C-EPDS) [28] was used to assess the criterion validity of the C-MAS. The EPDS consists of 10 items rated on a 4-point scale, with item scores ranging from 0 to 3 and total scores ranging from 0 to 30. The cutoff point for depression was set at 9.5; scores of 0 to 9 were considered normal, and scores of ≥10 indicated risk for depression. Higher scores indicate more severe depressive symptoms. The scale demonstrated a Cronbach’s α of .76 for reliability and a content validity index of .93 in previous research [28]. In this study, the reliability of the C-EPDS was confirmed with a Cronbach’s α of .82.

#### Infant-care social support

The Chinese version of the Infant Care Social Support Scale (C-PICSS) [29] was used to evaluate the criterion validity of the C-MAS. This scale consists of 19 items across three domains: information support, support systems, and practical support. Each item is rated on a 5-point Likert scale, with higher scores indicating higher levels of social support. The reliability of the C-PICSS was reported as Cronbach’s α=.93 in previous research [29] and Cronbach’s α=.97 in the present study.

#### Participants’ characteristics

Maternal and paternal demographic characteristics, health characteristics, and family-related variables were assessed. Demographic characteristics included age, employment status, education, and monthly family income. Health characteristics included the method of conception and mode of delivery, as well as maternal and infant health status. Family-related variables included marital relationship quality and husband’s involvement in household chores and childcare. These variables were measured to examine their relevance to maternal adaptation.

### Data collection

Data were collected from December 13, 2024, to March 21, 2025. The research team explained the study objectives and methods to outpatient nurses in charge of obstetrics, gynecology, and pediatrics at two hospitals in two different regions and requested their cooperation with recruitment. The first author posted participant recruitment flyers on hospital bulletin boards. Potential participants then met with the first author to verify eligibility and receive an explanation of the study aims and procedures. Mothers who agreed to participate accessed an online survey platform through a link, provided consent by selecting the “yes” option, and completed the study questionnaire. The questionnaire took approximately 15 minutes to complete. Participants received baby diapers or wipes as a token of appreciation.

### Data analysis

Data were analyzed using IBM SPSS ver. 26.0 and IBM SPSS AMOS ver. 26.0 (IBM Corp., Armonk, NY, USA), and Mplus ver. 8.3 (Muthén & Muthén, Los Angeles, CA, USA). Statistical significance was set at *p*<0.05. Item analysis was conducted by examining Spearman rho (ρ) correlations between each of the 28 individual items and the total score. The construct validity of the C-MAS was tested using confirmatory factor analysis (CFA) with the weighted least squares mean- and variance-adjusted (WLSMV) estimator in Mplus. Based on the characteristics of each fit index, model fit was evaluated using the normed chi-square (χ²/df), root mean square residual (RMR), standardized root mean square residual (SRMR), root mean square error of approximation (RMSEA), Tucker-Lewis index (TLI), and comparative fit index (CFI) [30,31]. Model fit was considered acceptable when χ²/df ≤3, RMR and SRMR <0.05, RMSEA <0.08, and TLI and CFI >0.90 [30,32].

To further examine the stability of the factor structure across parity groups, measurement invariance was tested using multigroup CFA in Mplus. Configural, metric, and scalar invariance models were evaluated. Because the items were measured on ordinal scales with fewer than five response categories, maximum likelihood estimation was considered inappropriate. In this context, the WLSMV estimator is designed for ordinal data and provides more accurate fit indices and standard errors [33]. Metric and scalar invariance were evaluated, and changes in CFI remained below .01 across models, satisfying the established criterion for measurement invariance [34]. If measurement invariance was supported, CFA was rerun using the full sample. Although normality is not required for ordinal data, skewness and kurtosis were evaluated descriptively in this study.

Convergent validity was assessed using standardized factor loadings, composite reliability (CR), and average variance extracted (AVE), with threshold values of 0.50, 0.60, and 0.50, respectively [35]. Discriminant validity was evaluated by comparing the square root of each construct’s AVE with its interconstruct correlations. Evidence of discriminant validity was considered present when the square root of the AVE exceeded the corresponding interconstruct correlations.

Criterion validity was examined using Pearson correlation analysis among maternal adaptation, parenting competence, infant-care social support, and postpartum depression, because these variables were treated as continuous. To evaluate known-groups validity, maternal adaptation levels were compared across groups defined by postpartum depression level. Reliability was further examined using item–total correlations and Cronbach’s α coefficients to evaluate the internal consistency of the C-MAS. Finally, independent t-tests and one-way analysis of variance were performed to identify differences in C-MAS scores according to participants’ demographic and obstetric characteristics. Scheffé post hoc procedures were used to adjust for multiple comparisons.

## Results

### Participants’ characteristics

The mean maternal age was 27.55±4.40 years, and 45.2% of participants were employed. The proportion of mothers with a college degree or higher was 21.6%. Only 23.2% of families had an average monthly income of 5,000 Chinese yuan (¥) or more, whereas 90.0% of participants subjectively rated their economic status as “above average.” Most participants had a natural pregnancy (76.6%) and vaginal birth (72.8%), and 77.2% reported breastfeeding or mixed feeding. Although 95.8% reported that their infants were in good health, 66.0% reported that their own health was good. Despite 96.0% of mothers reporting satisfaction with their marital relationship, only 52.0% reported above-average spousal help with household chores, and 43.2% reported above-average spousal participation in childcare. Among the 500 mothers, 268 (53.6%) were primiparous and 232 (46.4%) were multiparous (Table 1).

### Psychometric testing of the instrument

#### Item analysis

Item analysis was conducted by examining correlations between each of the 28 individual items and the total score. The items “I am afraid of taking care of the baby alone” and “I am inexperienced in taking care of the baby as a mother” were reverse-coded before analysis. Both items showed relatively low negative correlations with the total score (ρ=−.15 and ρ=−.16, respectively) (Supplementary Table 1). Because both items had item–total correlations well below the commonly accepted threshold of 0.30 and produced negative factor loadings in subsequent factor analysis, they were considered statistically and conceptually inconsistent with the construct and were therefore removed from the scale [36]. After these two items were removed, item–total correlation analysis was conducted for the remaining 26 items. Spearman rho coefficients ranged from .58 to .82 (*p*<.001) (Supplementary Table 2).

#### Confirmatory factor analysis

The measurement model of the C-MAS demonstrated good fit to the data. The chi-square test was significant (*p*<.001), and the normed chi-square (χ²/df) was 2.37, below the recommended threshold of 3, indicating acceptable fit. The SRMR was 0.03, indicating low residuals and good fit. Other fit indices also supported an acceptable model, with TLI=0.95 and CFI=0.95, indicating strong model fit. The RMSEA was 0.05, with a 90% confidence interval of 0.047–0.057, which was within the acceptable range. Overall, these indices supported the construct validity of the C-MAS measurement model (Table 2).

To examine whether the factor structure of the Maternal Adaptation Scale was stable across parity groups, CFAs were conducted separately for primiparous and multiparous mothers. For primiparous mothers, the model fit indices were χ²/df=1.87 (*p*<.001), SRMR=0.04, RMSEA=0.05, CFI=0.93, and TLI=0.92. For multiparous mothers, the model also showed good fit, with χ²/df=1.90 (*p*<.001), SRMR=0.04, RMSEA=0.06, CFI=0.94, and TLI=0.93. Multigroup CFA was then conducted to examine measurement invariance across parity groups. The configural model demonstrated acceptable fit, indicating that the six-factor structure was consistent across primiparous and multiparous mothers. The metric invariance model showed minimal change in CFI (ΔCFI<.001), supporting invariance of factor loadings across groups. Similarly, the scalar invariance model showed comparable fit (ΔCFI<.001), indicating that scalar invariance was supported across parity groups (Table 2).

To verify whether the item distribution across subdomains in the original instrument was also appropriate in this study, CFA was rerun using the full C-MAS sample. This analysis assessed model fit and the adequacy of factor loadings for each item within the following six subdomains: (1) role performance ability, (2) role cognition ability, (3) maternal acceptance, (4) physical and physiological recovery, (5) adaptation to family relationships, and (6) adaptation to social relationships. All items had factor loadings greater than 0.60, with most exceeding 0.70. The AVE for each subdomain ranged from 0.54 to 0.64, exceeding the recommended threshold of 0.50. These findings indicated that the latent variables explained a substantial proportion of variance in the observed variables and supported convergent validity. In addition, the square roots of the AVE ranged from 0.73 to 0.80 and were all greater than the interconstruct correlations, indicating adequate distinctiveness among the subdomains. CR values ranged from 0.80 to 0.91, all exceeding the recommended value of 0.70, indicating high internal consistency and stability across subdomains. The Cronbach’s α coefficient for the overall scale was .96. The reliability coefficients for the six subdomains were .84, .80, .91, .87, .82, and .84, respectively, confirming a high level of reliability (Table 3). The C-MAS showed a similar factor structure for primiparous and multiparous mothers.

#### Criterion validity

To verify the criterion validity of the C-MAS, its associations with parenting competence, postpartum depression, and infant-care social support were examined using Pearson correlation analysis. The scale was significantly correlated with parenting competence (r=.39, *p*<.001), postpartum depression (r=−.39, *p*<.001), and infant-care social support (r=.14, *p*<.001) (Tables 4, 5).

#### Known-groups validity

To evaluate known-groups validity, maternal adaptation levels were compared according to postpartum depression level. Depression levels were categorized into three groups based on depression scores: normal (0–9), mild depression (10–12), and severe depression (≥13). Maternal adaptation scores differed significantly among the three groups (F=79.25, *p*<.001). The Scheffé post hoc test indicated that mothers in the normal group had significantly higher maternal adaptation scores than those in the mild and severe depression groups. In addition, when the analysis was stratified by parity, maternal adaptation scores differed significantly across the three depression groups among both primiparous and multiparous mothers (Table 6).

#### Levels of and differences in maternal adaptation by participants’ characteristics

The mean total maternal adaptation score was 75.50±17.52, with scores ranging from 26 to 104. The mean scores (±SD) for the six subscales were 14.63±3.61, 8.39±2.29, 20.98±5.38, 14.29±3.79, 8.54±2.41, and 8.65±2.41, respectively. The correlation coefficients between the six C-MAS subdomains and the total score ranged from .80 to .92 (*p*<.001), indicating moderate to strong positive relationships between each domain and overall maternal adaptation (Tables 4, 5).

Maternal adaptation scores were higher among mothers who were employed, had a college degree or higher, reported a monthly income above ¥5,000, perceived their health as good, and were first-time mothers (*p*<.05). Regarding obstetric characteristics, maternal adaptation scores were higher among mothers with a natural pregnancy, vaginal birth, and breastfeeding or mixed feeding (*p*<.05). Regarding family relationships, maternal adaptation scores were higher among mothers who reported very good marital relationships, whose spouses consistently helped with housework, and whose spouses actively participated in childcare (*p*<.001) (Table 1).

## Discussion

This study translated and validated the C-MAS. The C-MAS demonstrated good structural validity, convergent validity, and internal consistency reliability, supporting its suitability for assessing maternal role adaptation among primiparous and multiparous Chinese women during the postpartum period.

The C-MAS demonstrated good reliability, validity, and cross-cultural construct stability, supporting its use for assessing maternal role adaptation among Chinese mothers. The overall Cronbach’s α coefficient for the C-MAS was .96, and α coefficients for the six subdomains ranged from .80 to .91. These values are broadly consistent with the reliability reported for the original Korean version, which showed an α of .87 for the total scale and α values ranging from .73 to .84 across the six subscales [12].

Although high reliability is desirable, a very high α coefficient may indicate potential redundancy among items. Future studies should examine overlapping item content, consider reducing redundancy, and report complementary reliability indices, such as CR and McDonald’s omega.

Although the original C-MAS included 28 items with two reverse-coded items, the reverse-coding procedure for these items was rechecked and confirmed to have been properly applied before item analysis. After this verification, the final C-MAS consisted of 26 items because two reverse-coded items assessing “fear” and “inexperience” were excluded owing to low and negative item–total correlations (r=−.15). Such negative correlations may reflect coding errors or respondent misinterpretation rather than poor item quality. Reverse-keyed items can distort factor structure, reliability, and validity [37], although these items may be conceptually relevant to maternal adaptation. Future studies should verify reverse coding procedures and refine item wording to reduce misinterpretation. This process underscores the importance of culturally sensitive adaptation in scale validation and shows how careful item analysis can strengthen both reliability and theoretical coherence.

The criterion validity of the C-MAS was consistent with previous studies showing that parenting competence and social support facilitate maternal adaptation, whereas postpartum depression is a major barrier [9-11]. Although the correlation with infant-care social support was low (r=.14), the association was statistically significant, suggesting that maternal adaptation is shaped by multiple psychological and contextual factors [9,10]. Social support may act indirectly by buffering stress and facilitating coping, and its direct contribution to maternal role adaptation may vary across cultural and family contexts [11,24]. In this study, mothers with supportive family environments, including positive marital relationships and husbands’ involvement in household chores and childcare, reported higher levels of maternal adaptation. These findings support prior evidence that family dynamics are central to psychological adaptation [29,38,39]. A positive marital relationship may promote greater husband involvement in household and childcare responsibilities, and spousal support may serve as a proximal resource that facilitates maternal adaptation by sharing caregiving responsibilities and alleviating postpartum depressive symptoms [29,39]. In addition, broader family support may function as a contextual resource by providing emotional and practical assistance [38]. Together with higher parenting competence, which may be strengthened by supportive environments, these findings suggest that maternal adaptation is shaped through both emotional pathways, such as reduced depressive symptoms, and functional pathways, such as enhanced caregiving capacity, within a multilayered family support system. Clinical interventions should prioritize family-centered approaches that promote husband involvement, reinforce family support, and enhance parenting competence to facilitate maternal adaptation and reduce postpartum depression.

The known-groups validity analysis showed that maternal adaptation scores were significantly higher among mothers without postpartum depression than among those with mild or severe depression, both in the overall sample and in analyses stratified by parity. This finding is consistent with previous studies showing that depressive symptoms negatively affect maternal role adaptation [12,20,40]. Moreover, the findings underscore the importance of screening for postpartum depression in clinical practice because depressive symptoms may hinder mothers’ adjustment to their maternal roles.

Unlike the original K-MAS, which assessed maternal adaptation across a broad 12-month postpartum period, the present study focused specifically on 4 to 6 months postpartum, a developmentally salient phase in which maternal role identity becomes more consolidated [12]. This approach aligns with Mercer’s Becoming a Mother Theory [1], which identifies this period as critical for maternal role development while recognizing that becoming a mother is a continuous and evolving process.

The C-MAS demonstrated robust measurement invariance across parity groups, supporting its validity for meaningful group comparisons. This finding is particularly important in the Chinese context because the two-child policy introduced in 2016 was followed by an increase in multiparous births [16-18]. In this study, primiparous mothers showed better maternal adaptation than multiparous mothers. Consistent with the current findings, previous research suggests that sleep quality is a critical determinant of postpartum depression in both groups, whereas social support may buffer the structural strain of multichild caregiving and household management among multiparous mothers, which may complicate maternal adaptation [20]. By including both primiparous and multiparous mothers, this study strengthens the generalizability of the instrument and contributes to refinement of the maternal adaptation construct in the Chinese context. These results also highlight the need for tailored support programs that sustain the motivation of primiparous mothers while addressing the stress and practical challenges experienced by multiparous mothers.

This study has several limitations. First, the tool was validated using convenience sampling, and participants were drawn from specific regions, which may limit the generalizability of the findings. The results should therefore be interpreted with caution and may not be representative of broader populations. Second, the study included only mothers who were raising their infants with a spouse, which may further limit the applicability of the findings to mothers in other family structures, such as single mothers. Third, the sample showed relatively limited variability in marital satisfaction and perceived economic status, which may have influenced certain C-MAS subdomains and should be considered when interpreting the findings. Future research should use stratified or randomized sampling across diverse geographic and socioeconomic regions and include mothers from diverse family backgrounds to improve representativeness and strengthen the robustness of maternal role adaptation assessments among Chinese mothers. Fourth, this study used a cross-sectional design and collected questionnaire data at a single time point, which precluded examination of the dynamic evolution of maternal adaptation. Future qualitative research could incorporate in-depth interviews and discussions with postpartum mothers to explore their subjective experiences, challenges, and needs during maternal role adaptation. Such work would provide a stronger theoretical and practical foundation for scale revision and for the development of more targeted intervention programs.

This study demonstrated that the C-MAS has strong structural validity, convergent validity, and internal consistency reliability, supporting its suitability for evaluating maternal role adaptation among primiparous and multiparous Chinese women during the postpartum period. The strong psychometric properties of the C-MAS provide empirical support for its use in clinical assessment, psychological care, and targeted health policy initiatives addressing the transitional needs of postpartum women.

## Figures and Tables

**Table 1.** Levels of maternal adaptation by participants’ characteristics in Chinese mothers (N=500)

Variables	Categories	n (%)/mean±SD	Maternal adaptation	t/F (*p*)
			Mean±SD	
Maternal age (year)		27.55±4.40		
Maternal employment	Yes	226 (45.2)	80.39±15.64	5.93 (<.001)
	No	274 (54.8)	71.46±17.99	
Maternal education	Middle school or less^a^	182 (36.4)	77.39±16.27	51.08 (<.001)
	High school^b^	210 (42.0)	68.04±16.90	b<a<c
	College graduate and above^c^	108 (21.6)	86.81±13.50	
Family’s monthly income (CNY)	<3,000^a^	134 (26.8)	71.50±16.79	25.57 (<.001)
	3,000–5,000^b^	250 (50.0)	72.28±17.45	a<b<d<c
	5,001–8,000^c^	52 (10.4)	89.01±10.91	
	>8,000^d^	64 (12.8)	85.43±14.53	
Perceived household economic level	High^a^	41 (8.2)	73.14±16.68	6.80 (0.001)
	Middle^b^	409 (81.8)	74.70±17.69	a<c, b<c
	Low^c^	50 (10.0)	83.98±14.52	
Pregnancy method	Natural pregnancy	383 (76.6)	79.51±16.29	10.17 (<.001)
	Artificial pregnancy	117 (23.4)	62.35±14.81	
Type of delivery	Vaginal birth	364 (72.8)	76.93±16.81	2.87 (.004)
	Cesarean birth	136 (27.2)	71.65±18.83	
Infant feeding methods	Breastfeeding or mixed feeding	386 (77.2)	80.10±15.81	13.32 (<.001)
	Bottle feeding	114 (22.8)	59.92±13.70	
Baby’s health	Good	479 (95.8)	75.13±17.56	-2.26 (.024)
	Not good	21 (4.2)	83.95±14.64	
Mothers’ postpartum health status	Good	330 (66.0)	82.67±14.99	15.52 (<.001)
	Not good	170 (34.0)	61.57±13.16	
Marital relationship with spouse	Good	480 (96.0)	78.03±16.73	4.40 (<.001)
	Not good	20 (4.0)	70.75±18.04	
Husband’s involvement in household chores	Above average	260 (52.0)	85.52±16.45	5.64 (<.001)
	Below average	240 (48.0)	73.76±17.13	
Husband’s involvement in parenting	Above average	216 (43.2)	82.97±18.70	4.49 (<.001)
	Below average	284 (56.8)	73.90±16.86	
Parity	Primipara	268 (53.6)	79.26±14.93	5.20 (<.001)
	Multipara	232 (46.4)	71.15±19.25	

The Scheffé post hoc test was used to correct for multiple comparisons. CNY: Chinese yuan (5,000 CNY is roughly 732 US dollars).

**Table 2.** Model fit indices for the confirmatory factor analysis of the Chinese version of the Maternal Adaptation Scale (N=500)

Models	χ²	df	χ²/df	*p*	SRMR	RMSEA	TLI	CFI	ΔCFI
Measurement model									
Modified model	673.36	284	2.37	<.001	0.03	0.05	0.95	0.95	-
Primiparous mothers	532.81	284	1.87	<.001	0.04	0.05	0.92	0.93	
Multiparous mothers	541.13	284	1.90	<.001	0.04	0.06	0.93	0.94	
Measurement invariance testing			-						-
Configural invariance	1,073.94	568		<.001	0.04	0.05	0.93	0.94	-
Metric invariance	1,087.41	588		<.001	0.04	0.05	0.93	0.94	<.001
Scalar invariance	1,108.23	608		<.001	0.04	0.05	0.93	0.94	<.001

CFI: Comparative fit index; ΔCFI: change in value between two CFIs; df: degree of freedom; RMSEA: root mean square error of approximation; SRMR: standardized root mean square residual; TLI: Tucker-Lewis index.

**Table 3.** Confirmatory factor analysis of the Chinese version of the Maternal Adaptation Scale (C-MAS) (N=500)

Factors	Variables	C-MAS						
		Mean±SD	F1	F2	F3	F4	F5	F6
1. Role performance ability	1. Feed my baby comfortably	2.62±1.06	.87					
	2. Hold my baby comfortably	3.04±0.86	.84					
	5. Provide stimulation for my baby’s growth and development, such as a mobile or massage	2.94±0.90	.65					
	3. Bathe my baby comfortably	2.97±0.84	.64					
	4. Put my baby to sleep comfortably	3.04±0.89	.60					
2. Role cognition ability	7. Know what my baby wants	2.77±0.89		.81				
	8. Recognize my baby’s signs of sickness or pain	2.83±0.90		.76				
	6. Know why my baby cries	2.79±0.91		.70				
3. Maternal acceptance	11. Feel good while breastfeeding because I feel that I am doing something for my baby	2.89±0.98			.83			
	15. Feel happy when my baby seems to respond to me	2.90±1.01			.80			
	10. Feel happy to be a mother now	3.06±0.95			.79			
	9. Feel needed by my baby	3.05±0.91			.78			
	12. Accept that taking care of my baby is difficult but part of my role as a mother	3.04±0.88			.77			
	14. Feel satisfied with my new role as a mother	3.02±0.92			.75			
	13. Think of my baby’s needs before my own needs	2.99±0.90			.74			
4. Physical & physiological recovery	16. Feel less tired from parenting	2.96±0.89				.78		
	19. Take breaks from time to time while parenting	2.78±0.95				.78		
	20. Feel that my body is recovering from pregnancy and childbirth	2.87±0.90				.76		
	17. Experience less parenting-related sleep disturbance	2.84±0.95				.74		
	18. Feel less wrist and back pain related to parenting	2.82±0.95				.71		
5. Adaptation to family relationships	23. Feel that my relationship with my husband is becoming deeper through parenting	2.72±0.97					.79	
	22. Receive help from my husband when I need it	2.91±0.91					.78	
	21. Share my opinions and feelings about parenting with my husband	2.90±0.90					.77	
6. Adaptation to social relationships	25. Can ask friends and relatives for help with parenting issues	2.89±0.93						.84
	24. Have friends or relatives with whom I can discuss parenting difficulties	2.85±0.90						.78
	26. Regularly share parenting information with other mothers	2.90±0.88						.77
Average variance extracted (AVE)			.54	.57	.61	.57	.61	.64
Discriminant validity (√AVE)			.73	.75	.78	.75	.78	.80
Construct reliability			.85	.80	.91	.87	.82	.84
Cronbach’s α		.96	.84	.80	.91	.87	.82	.84

F1: Role performance ability; F2: role cognition ability; F3: maternal acceptance; F4: physical and physiological recovery; F5: adaptation to family relationships; F6: adaptation to social relationships.

**Table 4.** Relationships among maternal adaptation and other variables in Chinese mothers (N=500)

Variables	Mean±SD	r (*p*)			
		1	2	3	4
1. Maternal adaptation	75.50±17.52	1			
2. Parenting competence	62.33±7.33	.39 (<.001)	1		
3. Postpartum depression	13.95±5.31	–.39 (<.001)	–.56 (<.001)	1	
4. Infant-care social support	61.15±19.19	.14 (<.001)	.42 (<.001)	–.70 (<.001)	1

**Table 5.** Intercorrelations among the C-MAS subscales and total score in Chinese mothers (N=500)

C-MAS subscales	Mean±SD	r (*p*)					
		S1	S2	S3	S4	S5	S6
S1. Role performance ability	14.63±3.61	1					
S2. Role cognition ability	8.39±2.29	.72	1				
		(<.001)					
S3. Maternal acceptance	20.98±5.38	.77	.68	1			
		(<.001)	(<.001)				
S4. Physical and physiological recovery	14.29±3.79	.56	.67	.76	1		
		(<.001)	(<.001)	(<.001)			
S5. Adaptation to family relationships	8.54±2.41	.70	.60	.76	.76	1	
		(<.001)	(<.001)	(<.001)	(<.001)		
S6. Adaptation to social relationships	8.65±2.41	.70	.61	.73	.74	.80	1
		(<.001)	(<.001)	(<.001)	(<.001)	(<.001)	
C-MAS total	75.50±17.52	.87	.80	.92	.88	.87	.85
		(<.001)	(<.001)	(<.001)	(<.001)	(<.001)	(<.001)

C-MAS: Chinese version of the Maternal Adaptation Scale.

**Table 6.** Differences in maternal adaptation by levels of postpartum depression among Chinese mothers (N=500)

Variables	Mean±SD			F	*p*	Scheffé post-hoc test
	Normal (n=98)^a^	Mild depression (n=56)^b^	Severe depression (n=346)^c^			
Maternal adaptation (total)	92.56±11.15	76.30±17.48	70.54±15.91	79.25	<.001	a>b>c
Primiparous mothers	91.06±11.95	81.57±12.22	74.79±13.94	34.65	<.001	a>b>c
Multiparous mothers	95.13±9.21	71.03±20.39	66.03±16.64	47.53	<.001	a>b>c

## Supplementary materials

Further details on supplementary materials are presented online (available at https://doi.org/10.4069/whn.2026.06.03).

Supplementary Table 1. Item–total correlations of the Chinese version of the Maternal Adaptation Scale (C-MAS) with 28 items (N=500)

Supplementary Table 2. Item–total correlations of the final Chinese version of the Maternal Adaptation Scale (C-MAS) with 26 items (N=500)
