# Supplementary material for: Cross-cultural adaptation and validation of the Chinese version of the Maternal Adaptation Scale: a methodological study
Source: Womens Health Nurs. 2026 Jun 30;32(2):127–38. doi: 10.4069/whn.2026.06.03 (PMC13346791; doi:10.4069/whn.2026.06.03)
Supplement: Supplementary Table 1. — Item–total correlations of the Chinese version of the Maternal Adaptation Scale (C-MAS) with 28 items (N=500) [file whn-2026-06-03-Supplementary-Table-1.pdf]

Supplementary Table 1. Item–total correlations of the Chinese version of the Maternal Adaptation Scale (C-MAS) with 28 items (N=500)

Items	ρ	*p*
1. Feed my baby comfortably	.82	< .001
2. Hold my baby comfortably	.77	< .001
3. Bathe my baby comfortably	.62	< .001
4. Put my baby to sleep comfortably	.58	< .001
6. Give my baby the stimulus for growth and development (mobile, massage etc.)	.62	< .001
8. Know the reason why my baby cries	.61	< .001
9. Know what my baby's wants	.71	< .001
10. Know my baby's sick painful symptoms	.66	< .001
11. Having a baby who needs me	.75	< .001
12. Happy to be a mother now	.76	< .001
13. While breastfeeding, I feel good because I feel like I'm doing something for my baby	.78	< .001
14. Taking care of my baby is hard, but I accept that it is my job as a mother	.74	< .001
15. Think of my baby's needs before my needs	.70	< .001
16. Satisfied with my new role as a mother	.72	< .001
17. I'm happy that my baby seems to be reacting to me	.76	< .001
18. Feel less tired from parenting	.75	< .001
19. Feel less sleep disturbance from parenting	.68	< .001
20. Feel less pain in my wrist and back from parenting	.66	< .001
21. Taking a break from time to time while parenting	.77	< .001
22. My body is recovering from pregnancy and childbirth	.71	< .001
23. Share my opinions and feelings about parenting with my husband	.73	< .001
24. My husband helps me when I need him	.74	< .001
25. Feel that my relationship with my husband is getting deeper thanks to parenting	.76	< .001
26. Have friends or relatives to discuss about parenting difficulty	.74	< .001
27. Can ask for help from friends and relatives regarding parenting issues	.78	< .001
28. Share information with other mothers regularly for parenting	.72	< .001
MAS_5R. I am afraid of taking care of the baby alone	–.15	< .001
MAS_7R. I am inexperienced in taking care of the baby as a mother	–.16	< .001

ρ indicates Spearman rho.

https://doi.org/10.4069/whn.2026.06.03
