# Supplementary material for: Cross-cultural adaptation and validation of the Chinese version of the Maternal Adaptation Scale: a methodological study
Source: Womens Health Nurs. 2026 Jun 30;32(2):127–38. doi: 10.4069/whn.2026.06.03 (PMC13346791; doi:10.4069/whn.2026.06.03)
Supplement: Supplementary Table 2. — Item–total correlations of the final Chinese version of the Maternal Adaptation Scale (C-MAS) with 26 items (N=500) [file whn-2026-06-03-Supplementary-Table-2.pdf]

Supplementary Table 2. Item–total correlations of the final Chinese version of the Maternal Adaptation Scale (C-MAS) with 26 items (N=500)

Items	ρ	*p*
1. Feed my baby comfortably	.81	< .001
2. Hold my baby comfortably	.77	< .001
3. Bathe my baby comfortably	.62	< .001
4. Put my baby to sleep comfortably	.58	< .001
5. Provide stimulation for my baby's growth and development, such as a mobile or massage	.64	< .001
6. Know why my baby cries	.62	< .001
7. Know what my baby wants	.72	< .001
8. Recognize my baby's signs of sickness or pain	.66	< .001
9. Feel needed by my baby	.75	< .001
10. Feel happy to be a mother now	.76	< .001
11. Feel good while breastfeeding because I feel that I am doing something for my baby	.78	< .001
12. Accept that taking care of my baby is difficult but part of my role as a mother	.73	< .001
13. Think of my baby's needs before my own needs	.71	< .001
14. Feel satisfied with my new role as a mother	.72	< .001
15. Feel happy when my baby seems to respond to me	.76	< .001
16. Feel less tired from parenting	.75	< .001
17. Experience less parenting-related sleep disturbance	.69	< .001
18. Feel less wrist and back pain related to parenting	.67	< .001
19. Take breaks from time to time while parenting	.77	< .001
20. Feel that my body is recovering from pregnancy and childbirth	.71	< .001
21. Share my opinions and feelings about parenting with my husband	.74	< .001
22. Receive help from my husband when I need it	.74	< .001
23. Feel that my relationship with my husband is becoming deeper through parenting	.76	< .001
24. Have friends or relatives with whom I can discuss parenting difficulties	.75	< .001
25. Can ask friends and relatives for help with parenting issues	.78	< .001
26. Regularly share parenting information with other mothers	.73	< .001

ρ indicates Spearman rho.

https://doi.org/10.4069/whn.2026.06.03
